# Comparing Explainable Machine Learning Approaches With Traditional Statistical Methods for Evaluating Stroke Risk Models: Retrospective Cohort Study

**DOI:** 10.2196/47736

**Published:** 2023-07-26

**Authors:** Sermkiat Lolak, John Attia, Gareth J McKay, Ammarin Thakkinstian

**Affiliations:** 1 Department of Clinical Epidemiology and Biostatistics, Faculty of Medicine Ramathibodi Hospital Mahidol University Bangkok Thailand; 2 Centre for Clinical Epidemiology and Biostatistics, School of Medicine and Public Health Hunter Medical Research Institute University of Newcastle New South Wales Australia; 3 Centre for Public Health, School of Medicine Dentistry and Biomedical Sciences Queen's University Belfast Belfast United Kingdom

**Keywords:** stroke, machine learning, risk prediction model, explainable artificial Intelligence, risk factor, cohort study, high-risk patient, hypertension

## Abstract

**Background:**

Stroke has multiple modifiable and nonmodifiable risk factors and represents a leading cause of death globally. Understanding the complex interplay of stroke risk factors is thus not only a scientific necessity but a critical step toward improving global health outcomes.

**Objective:**

We aim to assess the performance of explainable machine learning models in predicting stroke risk factors using real-world cohort data by comparing explainable machine learning models with conventional statistical methods.

**Methods:**

This retrospective cohort included high-risk patients from Ramathibodi Hospital in Thailand between January 2010 and December 2020. We compared the performance and explainability of logistic regression (LR), Cox proportional hazard, Bayesian network (BN), tree-augmented Naïve Bayes (TAN), extreme gradient boosting (XGBoost), and explainable boosting machine (EBM) models. We used multiple imputation by chained equations for missing data and discretized continuous variables as needed. Models were evaluated using C-statistics and *F*_1_-scores.

**Results:**

Out of 275,247 high-risk patients, 9659 (3.5%) experienced a stroke. XGBoost demonstrated the highest performance with a C-statistic of 0.89 and an *F*_1_-score of 0.80 followed by EBM and TAN with C-statistics of 0.87 and 0.83, respectively; LR and BN had similar C-statistics of 0.80. Significant factors associated with stroke included atrial fibrillation (AF), hypertension (HT), antiplatelets, HDL, and age. AF, HT, and antihypertensive medication were common significant factors across most models, with AF being the strongest factor in LR, XGBoost, BN, and TAN models.

**Conclusions:**

Our study developed stroke prediction models to identify crucial predictive factors such as AF, HT, or systolic blood pressure or antihypertensive medication, anticoagulant medication, HDL, age, and statin use in high-risk patients. The explainable XGBoost was the best model in predicting stroke risk, followed by EBM.

## Introduction

Cardiovascular disease, especially stroke, is a major cause of death globally. Many risk factors for stroke include nonmodifiable (eg, ethnicity, age, and sex) and modifiable risk factors (eg, hypertension [HT], diabetes mellitus [DM], dyslipidemia [DLP], smoking, and alcohol consumption) [1]. Improved understanding of disease prediction and risk stratification are active epidemiological research areas to help clinicians target preventive treatment to those most likely to benefit.

The American Heart Association or American Stroke Association defines ischemic and hemorrhagic stroke [2]. Ischemic stroke is defined as an episode of neurological dysfunction caused by focal cerebral, spinal, or retinal infarction. A hemorrhagic stroke is characterized by an intracerebral hemorrhage, which involves bleeding within the brain tissue (parenchyma) or ventricular system. This condition, which is not caused by trauma, encompasses instances of spontaneous parenchymal hemorrhages or those occurring following a brain infarction. The rapid development of neurological dysfunction symptoms is a defining consequence of this internal bleeding.

There are 2 common sources of ischemic stroke: atherosclerotic stroke and cerebral embolism [3], with the former being more common. Atherosclerosis within a significant cerebral blood vessel can vary in severity from small changes in diameter to severe stenosis that can cause clotting at the site of the atherosclerotic plaque leading to blood flow obstruction, causing a stroke [4]. While a cerebral embolism can originate from other regions of the body, sometimes as a consequence of atrial fibrillation (AF), the emboli travel and obstruct the distal cerebral arteries preventing brain tissue perfusion leading to ischemia.

There are multiple risk factors for stroke given the various pathological pathways involved [5]. The Framingham Stroke Risk Profile is a composite vascular risk score that predicts 10-year stroke risk based on 8 risk factors, that is, age, systolic blood pressure (SBP), antihypertensive therapy, DM, cigarette smoking, cardiovascular disease, AF, and left ventricular hypertrophy [6]. The INTERSTROKE consortia identified 10 modifiable risk factors associated with 90% of the stroke population-attributable risk [7,8]. HT is regarded as the most important modifiable risk factor for hemorrhagic stroke, while recent smoking, DM, apolipoproteins, and cardiac causes are more critical factors associated with ischemic stroke.

Risk or prognostic prediction models of stroke have been developed using conventional statistical methods (such as multiple logistic regression [LR] or Cox proportion hazard [CPH] models) based on linear relationships with the outcome measure, allowing for 2-way interactions between risk factors [5,6,9-11]. In reality, the interaction between risk factors may be more complex, of a higher order, or nonlinear. Machine learning (ML) models free from prior hypotheses have been recently used for disease prediction given their ability to better consider the interactions present, including nonlinear relationships [12]. However, the causal inference of these methods remains questionable, in particular, whether these ML models actually reflect the underlying relevant biology or simply improve prognostic performance.

Many ML models (eg, decision tree, tree ensembles, support vector machines, and neural networks) and deep learning approaches have been compared to conventional statistical models to assess their ability to detect nonlinear associations and multifaceted interactions [13-17]. Deep learning models are composed of multiple hidden layers that include millions of parameters without clear mechanistic meaning, representing “black-box” models with little transparency [18,19]. To address this shortcoming, explainable ML approaches have become popular by improving features such as understandability, comprehensibility, interpretability, explainability, and transparency [18]. Explainable models include Bayesian network (BN) and tree-augmented Naïve Bayes (TAN) models, both of which are probabilistic graphical models [20]. An explainable boosting machine (EBM) is based on a generalized additive model and is considered a ”glass-box” model given its improved transparency and interpretability [21]. These models excel in capturing complex relationships and dependencies among features, providing a more comprehensive understanding of the data structure and interplay between different risk factors when compared with the traditional statistical LR model. Furthermore, extreme gradient boosting (XGBoost) is considered a state-of-the-art approach for evaluating tabular data [22].

Therefore, this study used real-world cohort data and explainable ML models to identify risk factors for stroke occurrence in high-risk patients. The importance and ranking of risk factors were used as a proxy for explainability.

## Methods

### Study Design

This study is a retrospective cohort analysis of high-risk patients with stroke treated at Ramathibodi Hospital in Bangkok, Thailand, from January 2010 to December 2020. The study included patients aged 18 years or older with at least 1 diagnosis of HT, AF, DM, or DLP. Participants were excluded if they had a prior stroke at the initial hospital visit or had only 1 visit during the study period.

The patient cohort was identified from Ramathibodi Hospital's electronic database using the International Classification of Diseases, 10th Revision (ICD-10) codes for risk factors and clinical features, such as HT (I10-I16), DM (E08-E13), AF (I48), and DLP (E78). The primary end points of interest were the development of ischemic stroke (I63) and hemorrhagic stroke (I61), as indicated by their respective ICD-10 codes. The features and criteria used in this study can be found in Multimedia Appendix 1.

### Predictive Features and Outcome

Each patient was followed up until stroke occurrence, loss to follow-up, or censoring at study end (December 31, 2020). The latter 2 events were censored on their final visit or study end date, respectively.

Baseline study predictive features included age, sex, AF, HT, DM, DLP, SBP, plasma glucose (PG), serum creatinine, BMI, low-density lipoprotein and high-density lipoprotein (HDL), triglyceride level, and medications (antihypertensives, antiplatelets, oral hypoglycemics and insulin, statin and nonstatin lipid-lowering drugs, and anticoagulants). These baseline features were identified and retrieved when patients were first identified in our electronic medical records. The missing data in this study, assumed to be missing at random, were filled in using multiple imputation by chained equations via scikit-learn’s IterativeImputer [23,24]. The percentage of missing data and features used in multiple imputation by chained equations for each imputed variable are detailed in Table S1-S3 in Multimedia Appendix 1. Continuous data were categorized on the basis of previous literature to improve interpretation and as a requirement of the BN model [25]. Details of discretization are provided in Table S4 in Multimedia Appendix 1. We randomly separated the data by hospital numbers into development and test sets with a ratio of 80:20; each patient appeared in only 1 data set to maintain independence between the data sets. Characteristics of patients between the 2 data sets are comparable, see Table S5 in Multimedia Appendix 1.

### Model Construction

We compared model performance and explainability between LR, CPH, BN, TAN, XGBoost, and EBM. We normalized continuous variables and used recursive feature elimination to select features in the LR model, whereas feature selection in XGBoost and EBM included self-selecting features during node splitting [26]. We manually selected features in the BN and TAN based on stroke pathophysiology and considered the appropriate network structure.

Scikit-learn served as the ML library for LR and XGBoost, with hyperparameter tuning using grid and random searches with successive halving (HalvingGridSearchCV and HalvingRandomSearchCV) and assigned imbalance ratio as weights to counter imbalanced class effects. We extracted LR coefficients and XGBoost’s features’ importance together with Shapley Additive Explanations (SHAP) to represent their explainability [27]. We constructed EBM using the open-source package *InterpretML* (Microsoft) [21]. Variable and interaction effects were plotted to determine their impact on the outcome.

We built a BN using GeNIe Modeler (BayesFusion, LLC) software based on the known causal pathways of disease [28] and trained it using discretized data. TAN structures were also determined using the training data and GeNIe (BayesFusion, LLC) software. The architectural details of the BN and TAN are shown in Multimedia Appendix 2. Models were evaluated with C-statistics and *F*_1_-scores. C-statistics, or area under receiver operating characteristics curve (AUC-ROC), provide a measure of a models’ ability to accurately distinguish between positive and negative classes (0.5 being no predictive ability beyond chance and 1 being perfect prediction), while the *F*_1_-score represents a measurement of the balance between precision and recall in binary classification, which computes by harmonic mean between precision and recall.

### Ethical Considerations

The data were anonymized to ensure confidentiality and privacy protection. This study was approved by Human Research Ethics Committee, Faculty of Medicine Ramathibodi Hospital, Mahidol University (COA. MURA2021/255). The committee waived the need to obtain consent for the collection, analysis, and publication of the retrospectively obtained and anonymized data for this noninterventional study.

## Results

A total of 275,247 high-risk patients were included in this cohort, of whom 9659 (3.5%) experienced a stroke. Specifically, 7874 patients had an ischemic stroke, and 2427 patients had a hemorrhagic stroke. The patient cohort included 19,324 (7%) patients with AF, 98,836 (36%) with DM, 228,055 (83%) with DLP, and 211,430 (77%) with HT. Table 1 presents the baseline characteristics, revealing significant differences between the stroke and nonstroke groups for almost all variables, except for DLP (*P*=.70). The data set was divided into development and validation sets, comprising 220,198 and 55,049 patients, respectively.

**Table 1:** Cohort summary statistics.

				Stroke (N=9659)		Nonstroke (N=265,588)		*P* value	
Age (years), mean (SD)				64.7 (13)		58 (14.1)		<.001	
**Sex**									<.001
	Male, n (%)			5107 (0.05)		101,700 (0.95)			
	Female, n (%)			4552 (0.03)		168,440 (0.97)			
**Medication,** **n (%)**									
	**Antihypertensive medication**							<.001	
		Yes	4096 (0.03)		141,188 (0.97)				
		No	5563 (0.04)		124,400 (0.96)				
	**Hypoglycemic medication**							<.001	
		Yes	1659 (0.03)		59,150 (0.97)				
		No	8000 (0.04)		206,438 (0.96)				
	**Lipid-lowering medication (nonstatin)**							<.001	
		Yes	913 (0.02)		38,580 (0.98)				
		No	8746 (0.04)		227,008 (0.96)				
	**Statin medication**							<.001	
		Yes	3369 (0.03)		126,508 (0.97)				
		No	6290 (0.04)		145,370 (0.96)				
	**Antiplatelet medication**							<.001	
		Yes	2868 (0.05)		54,992 (0.95)				
		No	6791 (0.03)		217,387 (0.97)				
	**Anticoagulant medication**							<.001	
		Yes	622 (0.06)		10,015 (0.94)				
		No	9037 (0.03)		255,573 (0.97)				
**Vital signs, mean (SD)**									
	Systolic blood pressure (mm Hg)			138 (22.7)		133.6 (20.9)		<.001	
	Diastolic blood pressure (mm Hg)			77.5 (11.1)		78 (10)		.002	
	BMI (kg/m^2^)			25.2 (4.4)		25.5 (4.8)		<.001	
**Risk factors,** **n (%)**									
	**Atrial fibrillation**							<.001	
		Present	2591 (0.13)		16,733 (0.87)				
		Absent	7068 (0.02)		248,855 (0.98)				
	**Dyslipidemia**							.70	
		Present	8017 (0.04)		220,038 (0.96)				
		Absent	1642 (0.03)		45,550 (0.97)				
	**Hypertension**							<.001	
		Present	8936 (0.04)		202,494 (0.96)				
		Absent	723 (0.01)		63,094 (0.99)				
	**Diabetes mellitus**							<.001	
		Present	4202 (0.04)		94,634 (0.96)				
		Absent	5457 (0.03)		170,954 (0.97)				
**Laboratory values,** **mean (SD)**									
	Plasma creatinine (mg/dL)			1.2 (1.4)		1.14 (1.7)		.001	
	Blood sugar (mg/dL)			128.3 (64.2)		111.6 (46)		<.001	
	Hemoglobin A_1c_ (%)			6.7 (1.7)		6.4 (1.5)		<.001	
	Low-density lipoprotein (LDL) (mg/dL)			119.9 (43.2)		128.3 (41.1)		<.001	
	High-density lipoprotein (HDL) (mg/dL)			45.7 (13.5)		50.9 (14)		<.001	
	Triglyceride (md/dL)			142 (96.7)		136.1 (94.8)		<.001	

In terms of discriminative performance, the XGBoost model yielded the highest C-statistic (0.89, 95% CI 0.88-0.90) and *F*_1_-score (0.80), followed by EBM and TAN with C-statistics of 0.87 (95% CI 0.86-0.87) and 0.83 (95% CI 0.82-0.83), respectively. LR and BN models demonstrated similar performances, with C-statistics of 0.80 (95% CI 0.79-0.81). These results are presented in Table 2.

**Table 2:** Model performance of stroke risk prediction over a 10-year period.

Model	C-statistics (95% CI)	*F*_1_-score
Logistic regression	0.80 (0.79-0.81)	0.73
Bayesian network	0.80 (0.79-0.81)	0.73
Explainable boosting machine	0.87 (0.86-0.87)	0.78
XGBoost^a^	0.89 (0.88-0.90)	0.80
Tree-augmented Naïve Bayes	0.83 (0.82-0.83)	0.73

^a^XGBoost: extreme gradient boosting.

The LR model identified several factors significantly associated with stroke including AF (odds ratio [OR] 5.93, 95% CI 5.86-5.99), HT (OR 5.14, 95% CI 5.1-5.18), antihypertensive medication (OR 0.3, 95% CI 0.24-0.35), antiplatelets (OR 3.01, 95% CI 2.96-3.07), HDL (OR 0.76, 95% CI 0.74-0.78), and age (OR 1.31, 95% CI 1.29-1.33) as shown in Table 3. Based on feature importance ranking and SHA*P* values, the XGBoost model identified AF, SBP, HDL, PG, antihypertensive medication, HT, and antiplatelets as significant factors associated with stroke occurrence (Figure S1 in Multimedia Appendix 3). The EBM model identified PG, antihypertensive medication, SBP, HDL, HT, and AF as significant features, with interaction terms providing no additional predictive power (Multimedia Appendix 4). For the BN and TAN models, advanced age (>75 years) combined with AF were the strongest factors. Overall, AF, HT, and antihypertensive medication emerged as common significant factors across most models. Notably, AF was the strongest factor in the LR, XGBoost, BN, and TAN models. Receiver operating characteristic and precision-recall curves of each model are provided in Multimedia Appendix 5.

**Table 3:** Odds ratio (OR) of variables from multivariate logistic regression model.

			OR (95% CI)
**Categorical variable**			
	AF^a^	5.93 (5.86-5.99)	
	HT^b^	5.14 (5.1-5.18)	
	antiHT^c^	0.3 (0.24-0.35)	
	antiPL^d^	3.01 (2.96-3.07)	
	antiDM^e^	0.51 (0.43-0.6)	
	DLP^f^	1.86 (1.78-1.93)	
	Statin	0.61 (0.56-0.67)	
	antiDLP^g^	0.67 (0.59-0.75)	
	antiCoag^h^	0.68 (0.58-0.79)	
	isMale^i^	1.4 (1.36-1.44)	
	DM^j^	1.31 (1.25-1.36)	
**Continuous variable**			
	HDL^k^ (mg/dL)	0.76 (0.74-0.78)	
	Age (years)	1.31 (1.29-1.33)	
	PG^l^ (mg/dL)	1.31 (1.29-1.32)	
	Cr^m^ (mg/dL)	0.89 (0.87-0.91)	
	SBP^n^ (mmHg)	1.11 (1.09-1.13)	
	BMIcalc^o^ (kg/m^2^)	0.94 (0.92-0.96)	
	LDL^p^ (mg/dL)	1.04 (1.02-1.07)	
	TG^q^ (mg/dL)	0.97 (0.95-0.99)	

^a^AF: atrial fibrillation.

^b^HT: hypertension.

^c^antiHT: antihypertensive medication.

^d^antiPL: antiplatelet medication.

^e^antiDM: hypoglycemic medication.

^f^DLP: dyslipidemia.

^g^antiDLP: nonstatin lipid-lowering medication.

^h^antiCoag: anticoagulant medication.

^i^isMale: male.

^j^DM: diabetes mellitus.

^k^HDL: high-density lipoprotein.

^l^PG: plasma glucose.

^m^Cr: serum creatinine.

^n^SBP: systolic blood pressure.

^o^BMIcalc: body mass index.

^p^LDL: low-density lipoprotein.

^q^TG: triglycerides.

## Discussion

### Principal Findings

We investigated a retrospective cohort of patients at high risk of developing stroke to develop prediction models for stroke occurrence. The models identified AF, HT, or SBP or antihypertensive medication, anticoagulant medication, HDL, age, and statin use as important features in predicting stroke using both conventional LR and ML models. Our findings provide robust, transparent, and explainable ML models for stroke risk prediction using routinely collected clinical data accessible in general health care settings.

### Explainability

Explainability and transparency of risk prediction models are important for facilitating the prescribing of individualized treatments (precision medicine) in real-world clinical settings [29]. Improved patient understanding also leads to empowerment and improved medication or treatment adherence [30]. Improved understanding and use of ML models in both prehoc and post hoc analyses are growing. According to Arrieta et al [18], LR, CPH, BN, TAN, and EBM models are considered transparent and understandable in themselves, while XGBoost requires post hoc analysis to improve explainability, including local interpretable model-agnostic explanations [31], SHAP, partial dependence plots, feature importance [32], or DeepLIFT [17,18,33]. Explainability can be classified into 3 types: application-grounded, human-grounded, and functionality-grounded [34]. Application- and human-grounded categories involve human interpretability, that is, models that are easily comprehensible to a layperson, without the need for specialized technical knowledge or expertise; functionality grounded refers to the methods or algorithms used and their quantitative evaluation.

Some studies have explored the benefits of white-box ML prediction models, such as BN and EBM. For example, Park et al [35] used BN with a TAN algorithm to predict 3-month functional outcomes after stroke with an AUC-ROC of 0.889. Kanwar et al [36] used a BN-derived risk prediction model that improved the prediction of 1-year survival in patients with pulmonary arterial HT compared to the Kaplan-Meier method in REVEAL (version 2.0), with an AUC-ROC of 0.8 versus 0.76 [37,38]. Lou et al [39] showed that EBM approaches could achieve accuracy close to that provided by random forest models while providing good interpretability. White-box EBM approaches are also known as “glass-box” models that allow for interaction terms between variables within the model. All of these models performed well, and their white-box nature enables transparency, making them useful for clinicians to explain and translate medical knowledge for a more confident application in clinical settings. A previous study compared multiple ML models to predict stroke and address the class imbalance problem using a multilayer perceptron classifier to achieve the lowest false-negative rate (18.60%) and SHAP to investigate the impact of risk factors on stroke prediction [40]. However, this approach was considered a post hoc analysis and not representative of a white-box model.

To date, investigation of the predictive capabilities of multiple explainable models in the context of stroke risk assessment using real-world data has been limited. Our study addresses this knowledge gap through a novel approach that compares the performance metrics for several explainable models, resulting in significantly improved predictive accuracy, further informing the existing literature. ML models generally outperform traditional statistical methods, supported by AUC-ROCs that represent sufficient improvement to be clinically actionable, that is, AUC-ROCs over 0.80-0.85. This does not mean that all ML methods are superior to traditional statistical methods in all applications, and users should keep an open mind. Another benefit of transparent and explainable models is the rational and selective approach to the choice of predictors. Data mining methods used without regard to causative pathways can include variables that cause collider bias and reduce model performance or may lead to embedded bias within the observational data.

We seek to further contextualize our study findings in relation to the existing literature while also acknowledging the unique characteristics of our study population. The significant features we identified as contributing to stroke risks, such as AF and HT, have been extensively reported previously, providing validation of our findings [41]. However, it is important to note that our study offers additional insight into the strength and interaction of these risk factors to improve our understanding of stroke risk. They also show the potential to improve model performance over traditional approaches even when starting with an identical data set.

The integration of these models into clinical workflows could provide real-time, personalized risk assessments, guiding clinicians toward more targeted and effective interventions. For instance, a patient identified as high risk could be prioritized for aggressive preventive measures, such as rigorous lifestyle modification, counseling, or intensified medication regimens. Conversely, if a patient is predicted to have a lower stroke risk, they may avoid unnecessary treatments and potential side effects, or they might require less intensive follow-up within the hospital setting. This individualized approach would enhance the personalization of stroke prevention strategies, potentially improving patient outcomes.

In addition, the interpretability of the models used helps health care professionals to better understand the key drivers of the predicted stroke risk. This transparency could facilitate more informed and confident decision-making, bridging the gap between complex ML algorithms and their practical application in a clinical setting. Ultimately, these advances could lead to more efficient and effective personalized health care, underpinned by evidence-based, data-driven decisions.

### Limitations

There were several limitations to our study. First, other important epidemiological factors, such as smoking status, education, and alcohol consumption, were not included within our risk prediction models as the information was not recorded in the electronic medical records. Many variables rely on the accuracy of ICD-10 coding, which may be subject to miscoding or misdiagnosis that would reduce model performance. Our study cohort encompassed a somewhat narrow demographic range of an “at-risk” population. We recognize that stroke risk factors may vary across different populations, highlighting the need for externally validating our stroke prediction model in the future before wider application.

### Conclusions

Our study demonstrates predictive accuracy and explainability for stroke risk prediction models in high-risk patients. The key findings highlight the impact of AF, HT, and blood pressure control as significant risk factors for stroke emphasizing the potential benefits of screening and early detection, especially within patients for whom these risk factors are prominent. Furthermore, our findings confirm the robustness and interpretability of ML models such as XGBoost, EBM, and BN in handling complex, real-world health data and the potential to improve model performance even when starting with the same data set as traditional approaches.

Looking ahead, we anticipate significant opportunities for further research using these approaches. The continued evolution of ML techniques provides an avenue for refining prediction models, possibly by incorporating additional or alternative feature sets. Moreover, future studies could explore the effects of different interventions on stroke risk, such as lifestyle modifications or novel therapeutic agents. In doing so, our understanding of stroke prevention and management may be enhanced, potentially improving patient outcomes. By pushing the boundaries of explainable ML in health care, these findings hold the potential to revolutionize clinical practice, empowering physicians and patients with clear, actionable insights for better health outcomes.

## Appendix

Multimedia Appendix 1 Data management.

Multimedia Appendix 2 Bayesian network and tree-augmented Naïve Bayes.

Multimedia Appendix 3 Extreme gradient boosting.

Multimedia Appendix 4 Explainable boosting machine.

Multimedia Appendix 5 Receiver operating characteristic curve and precision-recall curve from baseline models.

## Abbreviations

AF: atrial fibrillation
AUC-ROC: area under receiver operating characteristics curve
BN: Bayesian network
CPH: Cox proportional hazard
DLP: dyslipidemia
DM: diabetes mellitus
EBM: explainable boosting machine
HDL: high-density lipoprotein
HT: hypertension
ICD-10: International Classification of Diseases, 10th Revision
LR: logistic regression
ML: machine learning
OR: odds ratio
PG: plasma glucose
SBP: systolic blood pressure
SHAP: Shapley Additive Explanations
TAN: tree-augmented Naïve Bayes
XGBoost: extreme gradient boosting

## Data Availability

Further use of data can be requested from the corresponding author.
