# Supplementary material for: Comparing Explainable Machine Learning Approaches With Traditional Statistical Methods for Evaluating Stroke Risk Models: Retrospective Cohort Study
Source: JMIR Cardio. 2023 Jul 26;7:e47736. doi: 10.2196/47736 (PMC10413234; doi:10.2196/47736)
Supplement: Multimedia Appendix 1 [file cardio_v7i1e47736_app1.docx]

## **Multimedia Appendix 1.** Data management.

**Table S1.** Variables used in the models.

| Variable | Description | Type | Definitive criteria / Icd-10 |
| --- | --- | --- | --- |
| age | Age | Continuous | - |
| isMale | Sex | Binary | Male |
| AF | Atrial fibrillation | Binary | I48 |
| HT | Hypertension | Binary | I10-I16 |
| DM | Type 1 and type 2 diabetes mellitus | Binary | E08-E13 |
| dlp | Dyslipidemia | Binary | E78 |
| antiHT | Antihypertensive medication | Binary | Receive med. |
| antiPL | Antiplatelets medication | Binary | Receive med. |
| antiDM | Hypoglycemic medication/Insulin | Binary | Receive med. |
| statin | Statin medication | Binary | Receive med. |
| antiDLP | Non-statin lipid lowering medication | Binary | Receive med. |
| antiCoag | Anticoagulant medication | Binary | Receive med. |
| SBP | Systolic blood pressure | Continuous | - |
| PG | Plasma glucose | Continuous | - |
| Cr | Serum creatinine | Continuous | - |
| BMIcalc | Body mass index | Continuous | - |
| LDL | Low-density lipoprotein | Continuous | - |
| HDL | High-density lipoprotein | Continuous | - |
| TG | Triglyceride | Continuous | - |
| isStroke | Stroke | Binary | I61, I63 |

**Table S2.** Missing values for each variable.

| Missing value: |  |
| --- | --- |
| Serum Creatinine (Cr) | 31587 (11%) |
| Triglyceride (TG) | 39109 (15%) |
| High-density Lipoprotein (HDL) | 72649 (26%) |
| Low-density Lipoprotein (LDL) | 64845 (24%) |
| Plasma Glucose (PG) | 43025 (16%) |
| Hemoglobin A1C (HbA1C) | 125011 (45%) |
| Body Mass Index (BMI) | 66205 (24%) |
| Systolic Blood pressure and Diastolic Blood Pressure (SBP, DBP) | 44633 (16%) |

**Table S3.** Variables uses in Multiple Imputation by Chained Equations imputation.

| Imputation parameter | |
| --- | --- |
| Cr | Age, sex, Hypertension, Antiplatelets, Antihypertensive medication, Diabetes, Hypoglycemic medication, Stroke, Uric acid, Body weight |
| SBP, DBP | Age, sex, Hypertension, Antiplatelets, Antihypertensive medication, Diabetes, Hypoglycemic medication, Stroke, Uric acid, Body weight |
| TG, HDL, LDL | Age, sex, Blood pressure, Plasma glucose, Statin medication, non-Statin lipid lowering medication, Antiplatelets, Stroke, Hypertension |
| PG | Age, sex, Hypoglycemic medication, Antiplatelets, Stroke, Body weight, Hypertension, Serum creatinine |
| Weight, Height | Age, sex, Plasma glucose, Lipid profiles, Hemoglobin, Blood pressure, Stroke |

| Variables | Value | | | |
| --- | --- | --- | --- | --- |
| Age (year) | >75 | 65-75 | 55-65 | 0-55 |
| Serum creatinine (mg/dL) | >=1.5 | 1.2-1.5 | 0-1.2 |  |
| Triglycerides (mg/dL) | >250 | 200-250 | 150-200 | 0-150 |
| High-density lipoprotein (mg/dL) | 0-40 | 50-60 | 40-50 | >60 |
| Low-density lipoprotein (mg/dL) | >160 | 130-160 | 100-130 | 0-100 |
| Plasma glucose (mg/dL) | >=140 | 110-140 | 0-110 |  |
| Systolic blood pressure (mmHg) | >140 | 130-140 | 120-130 | 0-120 |
| Diastolic blood pressure (mmHg) | >=90 | 80-90 | 0-80 |  |
| Body mass index (kg/m^2^) | >27 | 24.5-27 | 23-24.5 | 0-23 |
| Discretized value | **1** | **2** | **3** | **4** |

**Table S4.** Discretization for value of each variable.

**Table S5.** Summary statistics between training and test datasets.

|  | Training set  (N = 233,959) | Test set  (N = 41,288) | *P* |
| --- | --- | --- | --- |
| Age, years (Mean ± SD) | 58.3 ± 14.1 | 58.3 ± 14.2 | 0.55 |
| Sex |  |  | 0.23 |
| Male, N (%) | 90895 (0.39) | 15912 (0.39) |  |
| Female, N (%) | 143064 (0.41) | 25376 (0.41) |  |
| Medication, N (%) |  |  |  |
| Antihypertensive medication | 123571 (0.53) | 21713 (0.53) | 0.39 |
| Hypoglycemic medication | 51754 (0.22) | 9055 (0.22) | 0.40 |
| Lipid lowering medication (non-Statin) | 33760 (0.144) | 5733 (0.139) | 0.003 |
| Statin medication | 110459 (0.47) | 19418 (0.47) | 0.50 |
| Antiplatelet medication | 49242 (0.21) | 8618 (0.21) | 0.43 |
| Anticoagulant medication | 9040 (0.04) | 1597 (0.04) | 0.98 |
|  |  |  |  |
| Vital Signs |  |  |  |
| Systolic Blood Pressure,  mmHg (Mean ± SD) | 133.8 ± 20.9 | 133.5 ± 20.8 | 0.03 |
| Diastolic Blood Pressure,  mmHg (Mean ± SD) | 77.9 ± 10 | 77.8 ± 10 | 0.02 |
| Body Mass Index,  kg/m² (Mean ± SD) | 25.5 ± 4.4 | 25.5 ± 4.8 | 0.1 |
|  |  |  |  |
| Risk factors, N (%) |  |  |  |
| Atrial Fibrillation | 16436 (0.07) | 2888 (0.07) | 0.83 |
| Dyslipidemia | 193910 (0.83) | 34145 (0.83) | 0.37 |
| Hypertension | 179782 (0.77) | 31648 (0.77) | 0.40 |
| Diabetes Mellitus | 84048 (0.36) | 14788 (0.36) | 0.68 |
|  |  |  |  |
| Laboratory values, (Mean ± SD) |  |  |  |
| Plasma Creatinine, mg/dL | 1.14 ± 1.7 | 1.14 ± 1.7 | 0.97 |
| Blood sugar, mg/dL | 112.2 ± 46.8 | 112.3 ± 47.3 | 0.79 |
| HbA1C, % | 6.5 ± 1.5 | 6.5 ± 1.5 | 0.69 |
| Low-density Lipoprotein (LDL), mg/dL | 128 ± 41.3 | 128.1 ± 41.1 | 0.58 |
| High-density Lipoprotein (HDL), mg/dL | 50.7 ± 14 | 50.7 ± 14 | 0.86 |
| Triglyceride, md/dL | 136.3 ± 94.8 | 136.2 ± 95.4 | 0.75 |
|  |  |  |  |
| Stroke, N (%) | 8210 (0.04) | 1449 (0.04) | 1.0 |
