# Supplementary material for: Comparing Explainable Machine Learning Approaches With Traditional Statistical Methods for Evaluating Stroke Risk Models: Retrospective Cohort Study
Source: JMIR Cardio. 2023 Jul 26;7:e47736. doi: 10.2196/47736 (PMC10413234; doi:10.2196/47736)
Supplement: Multimedia Appendix 2 [file cardio_v7i1e47736_app2.docx]

## **Multimedia Appendix 2.** Bayesian network and tree-augmented Naïve Bayes.


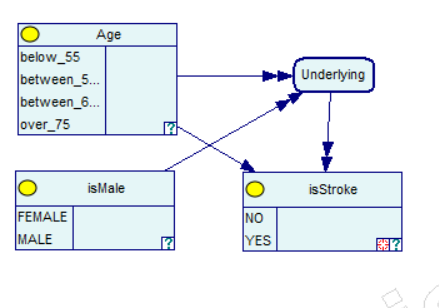


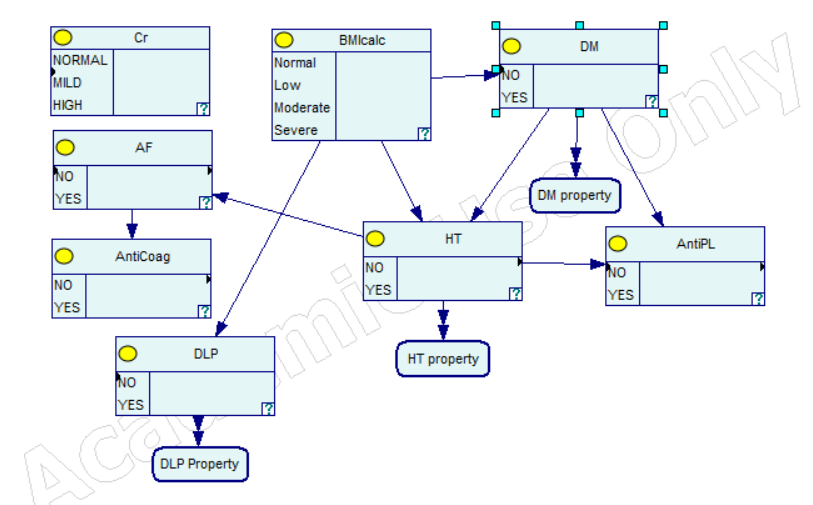


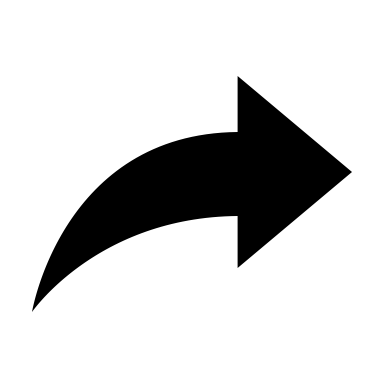


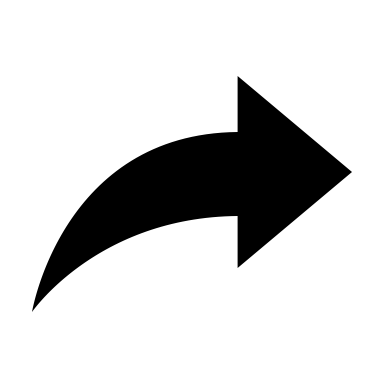


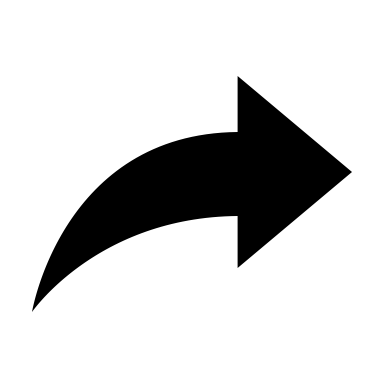


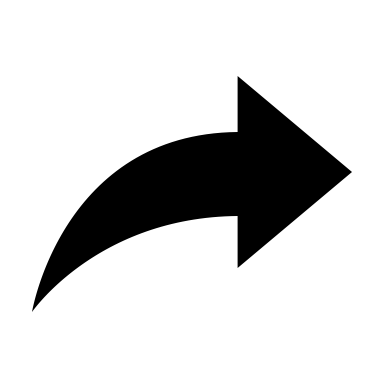


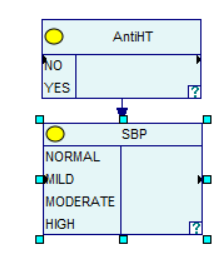

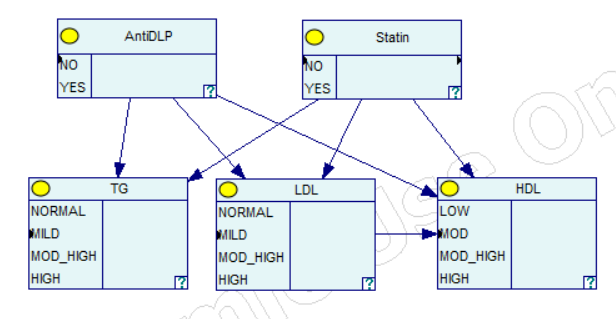


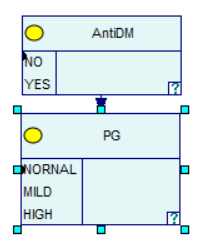


**Figure S1.** Bayesian Network architecture.

Each rectangle in the Bayesian network serves as a representation of a distinct variable, which is delineated categorically. The categories originate directly from the data or are determined through discretization if the data was initially presented in a numerical format. This process of discretization and the ranking of categories was based on severity as detailed further in Table 4. Nodes are encapsulated by a bold border and are representative of a node group, functioning as a container for ordinary nodes. Information regarding the parental and child relationships of each node can be found in Table S1.

**Table S1.** Summary of parents and children of each node in the Bayesian network.

| Node | Parents | Children |
| --- | --- | --- |
| BMIcalc | ‑ | DM, HT, DLP |
| Age | ‑ | DM, AF, Cr, DLP, AntiPL, isStroke |
| DM | BMIcalc, Age | HT, AntiDM, AntiPL, PG |
| isMale | ‑ | SBP, HDL |
| HT | DM, BMIcalc | AF, AntiHT, SBP, AntiPL, isStroke |
| AF | HT, Age | AntiCoag, isStroke |
| AntiCoag | AF | isStroke |
| Cr | Age | ‑ |
| AntiHT | HT | SBP, isStroke |
| DLP | BMIcalc, Age | Statin, AntiDLP, LDL, HDL, TG |
| SBP | HT, AntiHT, isMale | Stroke |
| Statin | DLP | LDL, HDL, TG, isStroke |
| AntiDM | DM | PG |
| AntiDLP | DLP | LDL, HDL, TG |
| LDL | DLP, Statin, AntiDLP | HDL |
| HDL | DLP, Statin, AntiDLP, LDL, isMale | ‑ |
| TG | DLP, Statin, AntiDLP | ‑ |
| AntiPL | Age, DM, HT | isStroke |
| PG | DM, AntiDM | ‑ |
| isStroke | AntiPL, SBP, AntiCoag, AF, Age, HT, AntiHT, Statin | ‑ |


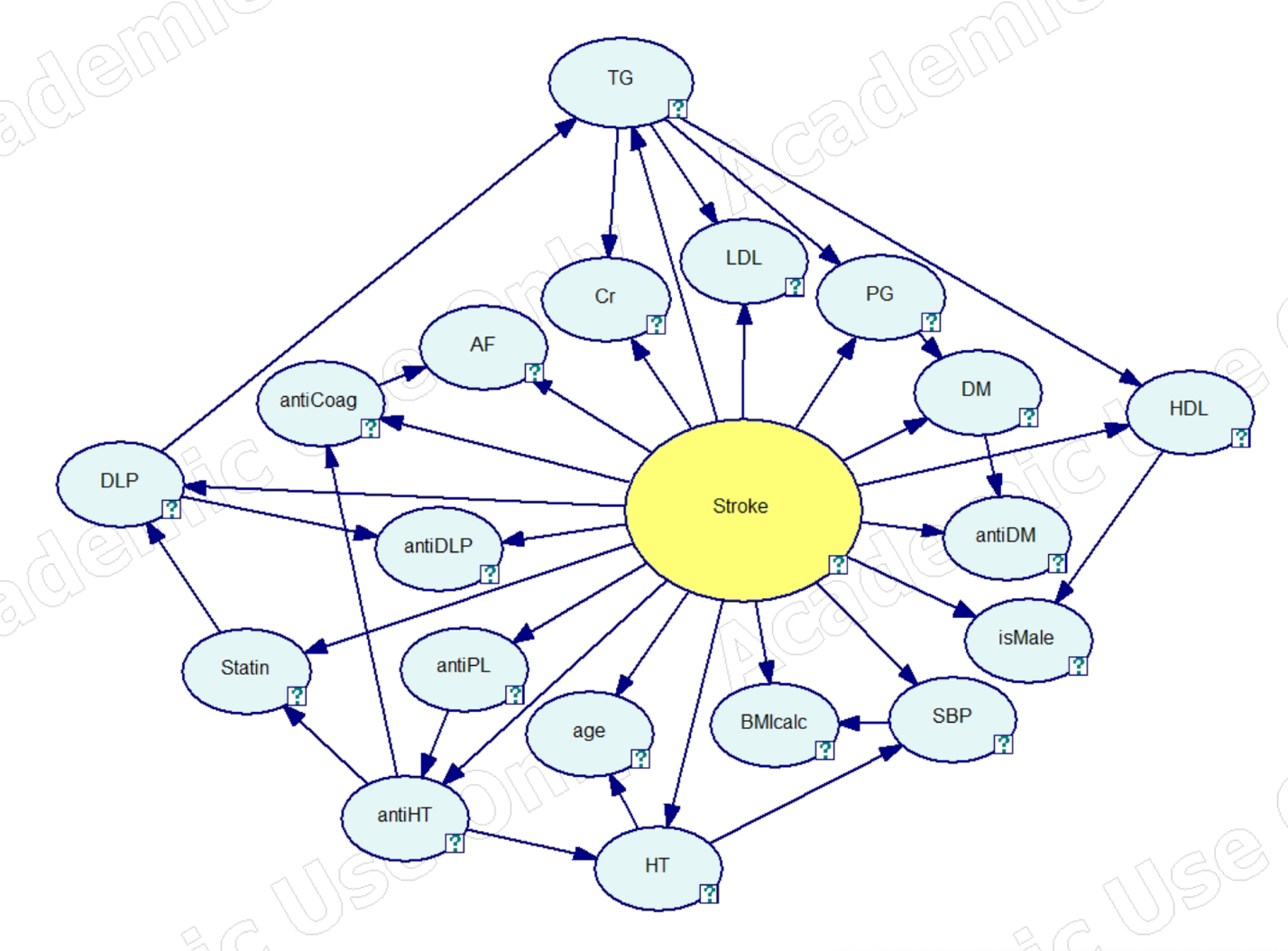


**Figure S2.** Tree-augmented Naïve Bayes architecture.
