# Supplementary material for: Comparing Explainable Machine Learning Approaches With Traditional Statistical Methods for Evaluating Stroke Risk Models: Retrospective Cohort Study
Source: JMIR Cardio. 2023 Jul 26;7:e47736. doi: 10.2196/47736 (PMC10413234; doi:10.2196/47736)
Supplement: Multimedia Appendix 3 [file cardio_v7i1e47736_app3.docx]

## **Multimedia Appendix 3.** Extreme gradient boosting.

**Table S1.** Parameters used for Scikit-learn’s XGBoostClassifier.

| Variable | Importance | Variable | Importance |
| --- | --- | --- | --- |
| AF | 0.240664 | **SBP** | 0.020101 |
| Statin | 0.131539 | **HDL** | 0.017186 |
| isMale | 0.129508 | **antiCoag** | 0.01676 |
| HT | 0.129121 | **PG** | 0.015928 |
| antiHT | 0.077227 | **DM** | 0.01338 |
| antiPL | 0.070552 | **Cr** | 0.009706 |
| antiDLP | 0.048903 | **DLP** | 0.006234 |
| antiDM | 0.042626 | **BMIcalc** | 0.003113 |
| age | 0.0223 | **LDL** | 0.0026 |
|  |  | **TG** | 0.002551 |

AF - Atrial fibrillation; Statin – Statin medication; isMale – Male; HT - Hypertension; antiHT - Antihypertensive medication; antiPL - Antiplatelets medication; antiDLP – non-Statin lipid lowering medication; antiDM - Hypoglycemic medication; SBP – Systolic blood pressure; HDL - High density lipoprotein; antiCoag – Anticoagulant medication; PG - Plasma Glucose; DM – Diabetes Mellitus; Cr – Serum Creatinine; DLP – Dyslipidemia; BMIcalc – Body Mass Index; LDL – Low density lipoprotein; TG – Triglycerides

**Figure S1.** SHapley Additive exPlantions (SHAP)'s features value.

**
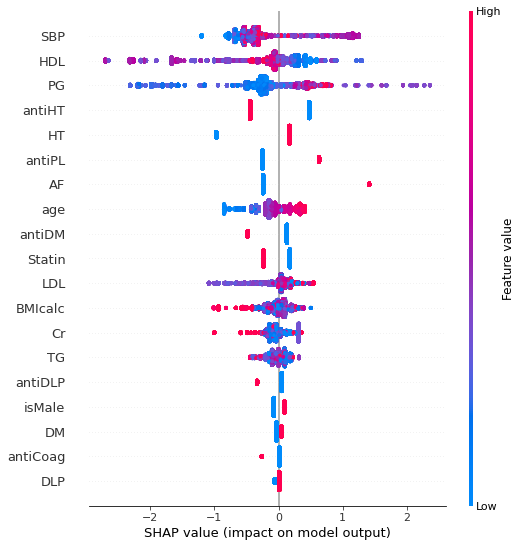
**

SBP – Systolic blood pressure; HDL – High density lipoprotein; PG – Plasma Glucose; antiHT – Antihypertensive medication; HT – Hypertension; antiPL – Antiplatelets medication; AF – Atrial fibrillation; antiDM – Hypoglycemic medication; Statin – Statin medication; LDL – Low density lipoprotein; BMIcalc – Body Mass Index; Cr – Serum Creatinine; TG – Triglycerides; antiDLP – non-Statin lipid lowering medication; isMale – Male; DM – Diabetes Mellitus; antiCoag – Anticoagulant medication; DLP – Dyslipidemia

## A SHAP summary plot provides a visualization of feature importance as well as the directionality of the relationship between each feature and the target variable. Each point on the graph represents a Shapley value on x-axis and a feature on y-axis. The color represents the value of the feature from low to high. Overlapping points are jittered in the y-axis direction for the distribution of the SHAP values per feature. The features are ordered according to their importance, which is determined by the sum of the SHAP value magnitudes across all instances. The most important feature is located at the top of the plot, which is represented by SBP, HDL, PG, antiHT, HT, and AF
