# Supplementary material for: Comparing Explainable Machine Learning Approaches With Traditional Statistical Methods for Evaluating Stroke Risk Models: Retrospective Cohort Study
Source: JMIR Cardio. 2023 Jul 26;7:e47736. doi: 10.2196/47736 (PMC10413234; doi:10.2196/47736)
Supplement: Multimedia Appendix 4 [file cardio_v7i1e47736_app4.docx]

**Multimedia Appendix 4.**
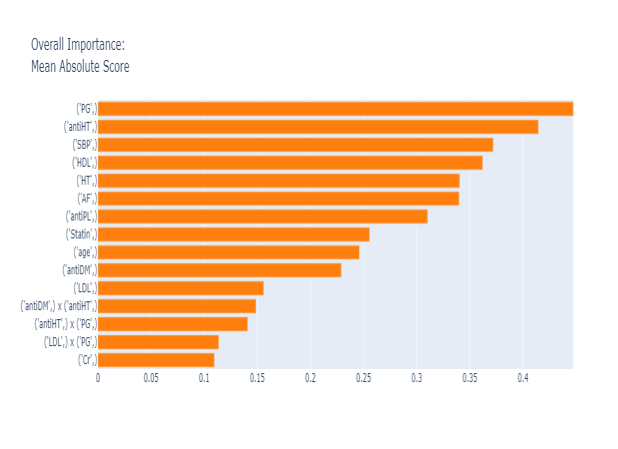
Explainable boosting machine

Figure 1. EBM's Features Importance. PG - Plasma Glucose; antiHT - Antihypertensive medication; SBP – Systolic blood pressure; HDL - High density lipoprotein; HT - Hypertension; AF - Atrial fibrillation; antiPL - Antiplatelets medication; Statin - Statin lipid lowering medication; antiDM - Hypoglycemic medication; LDL – Low density lipoprotein; Cr – Serum Creatinine
