# Supplementary material for: Comparing Explainable Machine Learning Approaches With Traditional Statistical Methods for Evaluating Stroke Risk Models: Retrospective Cohort Study
Source: JMIR Cardio. 2023 Jul 26;7:e47736. doi: 10.2196/47736 (PMC10413234; doi:10.2196/47736)
Supplement: Multimedia Appendix 5 [file cardio_v7i1e47736_app5.docx]

## **Multimedia Appendix 5.** Receiver operating characteristic curve and precision-recall curve from baseline models.

| 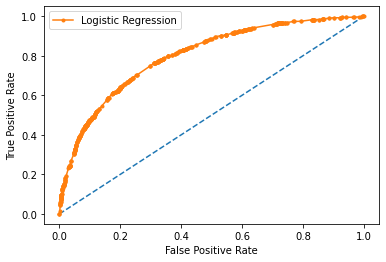  **Figure S1.** Logistic Regression (LR) ROC curve. | 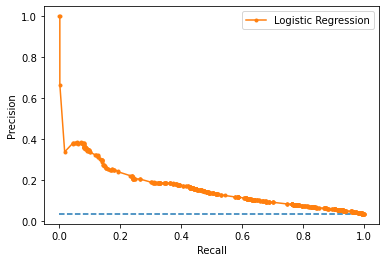  **Figure S2.** LR PR curve. |
| --- | --- |
| 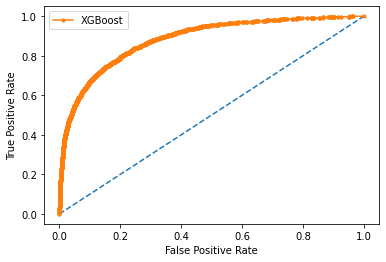  **Figure S3.** XGBoost ROC curve. | 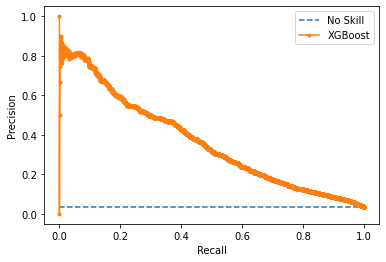  **Figure S4.** XGBoost PR curve. |

| 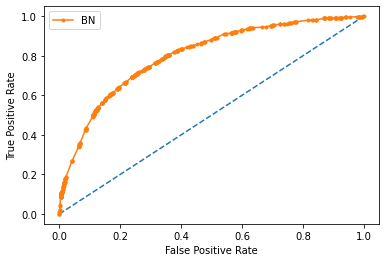  **Figure S5.** Bayesian network (BN) ROC curve. | 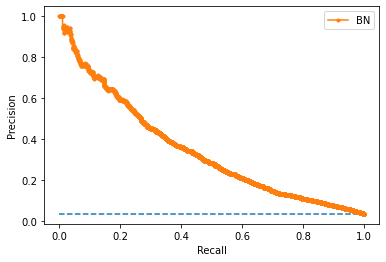  **Figure S6.** BN PR curve. |
| --- | --- |
| 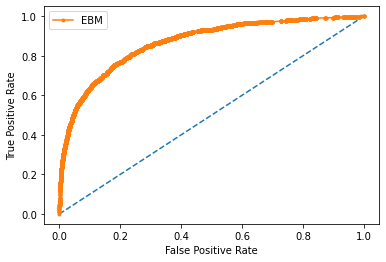  **Figure S7.** Explainable boosting machine (EBM) ROC curve. | 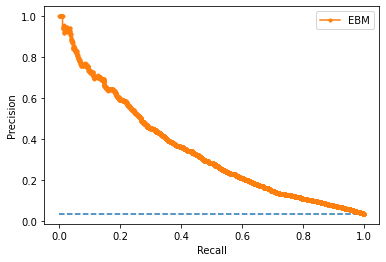  **Figure S8.** EBM PR curve. |
